# Identification of hippocampal cortical microinfarcts on postmortem 3-T magnetic resonance imaging

**DOI:** 10.1007/s00234-021-02717-8

**Published:** 2021-04-28

**Authors:** Max Scheffler, Rares Salomir, Enrique Maturana, Marie-Louise Montandon, Enikö V. Kövari, Sven Haller

**Affiliations:** 1 Division of Radiology, Geneva University Hospitals, Chemin du Pont-Bochet 3, 1226 Thonex, Switzerland; 2 Image Guided Interventions Laboratory, University of Geneva, Rue Gabrielle-Perret-Gentil 4, 1205 Geneva, Switzerland; 3 Division of Radiology, Geneva University Hospitals, Rue Gabrielle-Perret-Gentil 4, 1205 Geneva, Switzerland; 4 Department of Rehabilitation and Geriatrics, Geneva University Hospitals, Route de Loëx 151, 1233 Bernex, Switzerland; 5 Department of Psychiatry, Geneva University Hospitals, Chemin du Petit Bel-Air 2, 1225 Chene-Bourg, Switzerland; 6 CIMC - Centre d’Imagerie Médicale de Cornavin, Place de Cornavin 18, 1201 Geneva, Switzerland; 7 Radiology, Department of Surgical Sciences, Uppsala University, 75236 Sjukhusvägen, Sweden

**Keywords:** Cerebral microinfarcts, Hippocampus, Dementia, 3-T post-mortem MRI, Histopathology

## Abstract

Cortical microinfarcts (CMI) are increasingly recognized in the neurological community as a biomarker related to cognitive impairment and dementia. If their radiological depiction has been largely described in experimental settings using ultra-high-field magnetic resonance imaging (MRI), less is known about their visibility on routinely used 3-T MRI. In this radiologic-pathologic correlation study, using 3-T post-mortem MRI, we searched for hippocampal CMI, in a double-blinded fashion, and found that only 4/36, or 11%, were clearly demonstrated on both radiological and histopathological exams.

## Background

Cortical microinfarcts (CMI) correspond to small ischemic events of 0.05–4 mm in size, mostly measuring ≤ 1 mm [1]. They are associated with ß-amyloid deposits (57.1% of cases in one study [2]) and, depending on the affected brain region, atherosclerotic microangiopathy [2]. They are increasingly recognized in the neurological community as a radiological biomarker of vascular cognitive impairment [3, 4]. One meta-analysis of 32 neuropathological studies [5] found CMI in 62% of patients with vascular dementia (VaD), in 43% of patients with Alzheimer’s disease (AD), and in 33% of patients with mixed AD and VaD, compared to 24% of non-demented older individuals. Two in vivo magnetic resonance imaging (MRI) studies in the context of VaD found CMI in 20% and 32% of patients respectively [6, 7].

CMI of the hippocampal region were detected in an autopsy study in 44% of a total of 57 subjects with AD and cerebral infarcts [8]. Equally, over 50% of patients with vascular dementia showed hippocampal lesions in a neuropathological study of 20 cases [9].

The hippocampi are known to lie in a watershed territory between the internal carotid (anterior choroidal artery) and posterior cerebral artery perfusion territories, and the CA1 (CA, cornu ammonis) subfield is of particular vulnerability to ischemic events. The question was thus raised if the occurrence of CMI could be an indicator of hypoperfusion in the hippocampi [10].

Radiologically, CMI depiction was described in experimental settings by Van Veluw et al. [2] using post-mortem ultra-high-field MRI, but also more recently using 3-T MRI, despite their little size, using extended acquisition times of for example 1 h and 52 min for a T2-weighted sequence [11].

In our radiologic-pathologic correlation study using 3-T post-mortem MRI, we aimed to determine the number of hippocampal CMI that could be seen on both radiological and histopathological exams, using a double-blinded radiologic-pathologic approach.

## Material and methods

Post-mortem whole-brain 3-T MRI (Magnetom Skyra, Siemens, Erlangen, Germany) using a 64-channel head coil was performed on autopsy specimens of 21 patients. All brain specimens were prepared and scanned following a technique described earlier [12], including a T2-weighted 3D sequence that was used for the radiological analysis in this study by one neuroradiologist (M.S.), in line with previous reports on the MRI detection of CMI [13]. Acquisition parameters were as follows: TR, 1400 ms; TE, 158 ms; bandwidth, 289 Hz/pixel; flip angle, 120°; matrix size, 384 × 384 pixels; voxel size, 0.1 mm^3^; acquisition time, 20 min 25 s. CMI were defined as focal and well-delineated spots of elevated signal intensity in any of the three portions of the right hippocampus, first identified on a reconstructed coronal oblique plane and then confirmed on an orthogonal axial plane.

Secondly, the entire hippocampi of the right hemisphere were embedded in paraffin and cut into 50-µm-thick coronal-oblique slices, perpendicular to the long axis of the hippocampal corpus. Each 20th slice was stained with cresyl violet (Nissl stain). The slides were examined by a neuropathologist (E.V.K.) to identify CMI. The neuropathologist’s results, considered the gold standard, were blinded as referred to the radiologist’s findings. Consensus reading was performed thereafter, for comparison of images positivized by both readers.

## Results

**Fig. 1:**
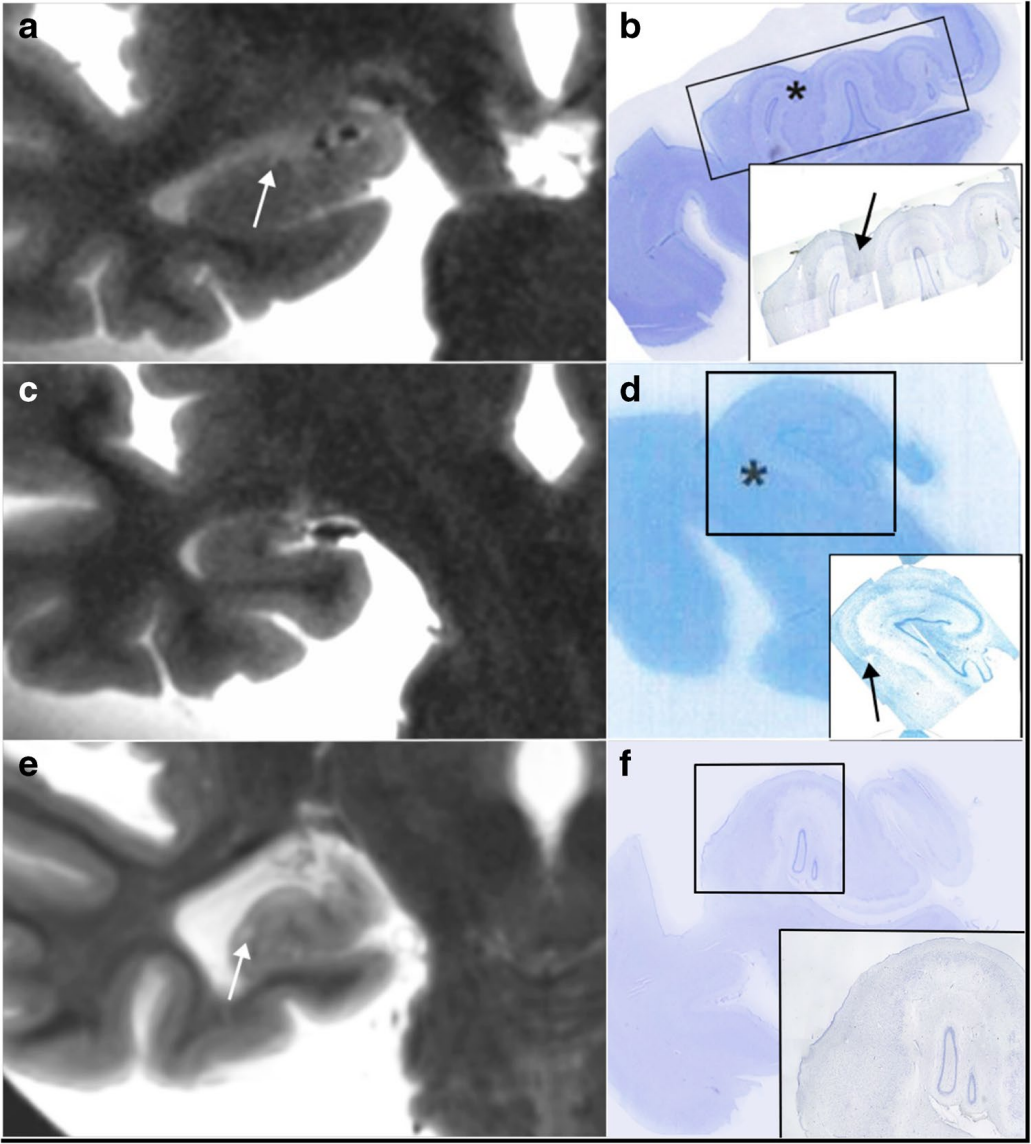
Post-mortem 3-T magnetic resonance imaging (MRI) T2-weighted image in coronal reconstruction shows millimetric signal hyperintensity in right hippocampal head (**a**, arrow), due to a cortical microinfarct (CMI) on the corresponding Nissl-stained histopathological image (**b**, asterisk on the main image, arrow on the inset). False negative MRI image (**c**) shows homogenous right hippocampal tail, without depiction of histopathologically evident CMI (**d**, asterisk on the main image, arrow on the inset). 3-T MRI T2-weighted image in coronal reconstruction shows focal signal hyperintensity in right hippocampal body (**e**, arrowhead), without a correlate on the subsequent histopathological exam (**f**). The image was considered a false positive finding

The results are presented in Tables 1, 2, and 3. In total, 36 CMI of the right hippocampus were found on the Nissl-stained slides of 21 autopsy exams, only 4 of which had a correlate on 3-T MRI, corresponding to a radiological true positive rate of 11%. No larger infarcts were present in the resected right-sided hippocampi. Ten right hippocampal signal hyperintensities on 3-T MRI remained without a histopathological correlate and were considered radiological false positives. Radiological sensitivity, specificity, and accuracy were 11.1%, 23.1%, and 2%, respectively. Illustrative examples of true positive, false negative, and false positive CMI after radiologic-pathologic consensus readings are provided in Fig. 1.

**Table 1:** Table shows histopathological and post-mortem 3-T MRI findings of the right hippocampus in 21 brain autopsies. The third column indicates all radiological true positive lesions

Patient no	CMB histopatho-logical exam (N)	Of which: visible and confirmed CMI on 3-T MRI (TP)	Suspected CMI on 3-T MRI, not confirmed (FP)	Radiological false negative (FN)	Radiological true negative (TN)
1	0	0	1	0	0
2	0	0	0	0	1
3	0	0	1	0	0
4	0	0	0	0	1
5	1	0	0	1	0
6	1	0	0	1	0
7	4	0	0	4	0
8	2	0	1	2	0
9	1	0	0	1	0
10	0	0	1	0	0
11	0	0	1	0	0
12	0	0	1	0	0
13	0	0	0	0	1
14	4	2	0	2	0
15	0	0	1	0	0
16	3	0	0	3	0
17	5	0	2	5	0
18	0	0	1	0	0
19	7	1	0	6	0
20	5	0	0	5	0
21	3	1	0	2	0
Total N	36	4	10	32	3

*CMI*, cortical microinfarct; *MRI*, magnetic resonance imaging; *TP*, true positive; *FP*, false positive; *FN*, false negative; *TN*, true negative

**Table 2:** A 2 × 2 table showing relation of radiological and histological findings

	MRI CMI negative	MRI CMI positive
Histological CMI negative	3	10
Histological CMI positive	32	4

*CMI*, cortical microinfarct; *MRI*, magnetic resonance imaging

**Table 3:** A 3 × 3 table summarizing findings of Tables 1 and 2 to show sensitivity, specificity, and accuracy of 3-T MRI to detect hippocampal CMI, with histological analysis presenting the reference standard

	Reference standard Histological CMI positive	Reference standard Histological CMI negative	
Index test MRI CMI positive	4 (TP)	10 (FP)	PPV 28.6%
Index test MRI CMI negative	32 (FN)	3 (TN)	NPV 8.6%
	Sensitivity 11.1%	Specificity 23.1%	Accuracy 2.0%

*CMI*, cortical microinfarct; *MRI*, magnetic resonance imaging; *TP*, true positive; *FP*, false positive; *FN*, false negative; *TN*, true negative; *PPV*, positive predictive value; *NPV*, negative predictive value

## Discussion

In the neurological community, an increasing interest is paid to CMI in patients suffering from cognitive decline in a known context of VaD, AD, or mixed dementia (AD plus VaD). With the hippocampi constituting a region of particular interest for cognitive decline and dementia, we decided to focus our study on lesions of this organ. Our work on 21 patients showed CMI to be of limited visibility on post-mortem 3-T MRI with imaging parameters resembling clinical routine imaging, with a radiological true positive rate of only 11% after radiologic-pathologic correlation. This might be attributed to small CMI size in our series, far below an accepted upper limit of 4 mm, and approaching the limits of spatial resolution on 3-T MRI. On the other hand, 7-T MRI studies showed promising results in detecting small CMI [11], but analyses were not focused so far on the hippocampal region. The important question of the correlation between CMI size and clinical repercussion is beyond the scope of this report but needs to be further clarified.

In addition to the limited number of true positives on 3-T MRI, we found a significant number of radiological false positives (10/21), thought to be due to superficial preparation-related lesions in the brain specimens and small hippocampal remnant cysts (between the dentate gyrus and the cornu ammonis). As a consequence, overall performance of the method was low, with an accuracy of only 2%.

Contrarily to earlier studies, we used a double-blinded approach between the radiologist and the pathologist, instead of using MRI-detected lesions as targets for histological analysis. We also opted for a shorter imaging time as compared to previous reports, closer to routine scanning of alive patients, as our main interest was clinical applicability in the case of positive results.

Several limitations of the study should be noted. If the detectability of microinfarcts is already low for 1.5-T and 3-T MRI in the hippocampal region, small physiologic cysts (of the abovementioned type) are frequent lesions that may cause further false positive findings. Another limitation lies in the lack of correlation between in vivo and post-mortem MRI, where future studies will have to further investigate if the process of formalin fixation changes the volume of small cystic lesions within the brain parenchyma. Finally, it has to be kept in mind that even the histopathological exam, considered the gold standard, remains incomplete. In our study only each 20th slice of the right hippocampus underwent analysis, and it cannot be excluded that small CMI lesions remained unseen in the 1-mm interslice gaps. In a future study with a similar design to ours, a systematic “second look” approach might be useful, where all lesions positivized by MRI are subject to novel analysis of additional “between gaps” histopathological slices.

In conclusion, CMI are increasingly recognized to in dementia research, possibly contributing independently to cognitive decline. Post-mortem 3-T MRI does not seem to be an adequate technique for the detection of these lesions when located in the hippocampi. Moreover, false positive findings might be secondary to small superficial defects occurring during specimen preparation, or small physiologic cysts frequently found in this region.
